# Endoscopic Submucosal Dissection of Large Gastric Hyperplastic Polyps: Clinicopathological Study From a Single Center

**DOI:** 10.7759/cureus.88911

**Published:** 2025-07-28

**Authors:** Xiaobang Hu, Julie C Fanburg-Smith, Francesca M Ruggiero, Justin B Roy, Robert Pantaleon Vasquez, Matthew T Moyer, Abraham Mathew

**Affiliations:** 1 Department of Pathology and Laboratory Medicine, Penn State Health Milton S. Hershey Medical Center, Penn State University College of Medicine, Hershey, USA; 2 Department of Pathology, University of Virginia School of Medicine, Charlottesville, USA; 3 Department of Gastroenterology, WellSpan Health, Lebanon, USA; 4 Department of Pathology, Hartford Hospital, Hartford, USA; 5 Division of Gastroenterology and Hepatology, Penn State Health Milton S. Hershey Medical Center, Penn State Cancer Institute, Hershey, USA; 6 Division of Gastroenterology and Hepatology, Penn State Health Milton S. Hershey Medical Center, Hershey, USA

**Keywords:** endoscopic mucosal resection, endoscopic submucosal dissection, field effect, gastric hyperplastic polyp, intraoperative lateral margin ink, recurrence

## Abstract

Background

Gastric hyperplastic polyps (GHPs) represent a subset of gastric polyps that are clinically problematic when large and they can cause persistent, low-grade bleeding or gastric outlet obstruction. Rare malignant transformation has been reported, albeit not in our experience.

Methods

We retrospectively studied the clinicopathologic characteristics of large GHPs (≥20 mm) excised by endoscopic submucosal dissection (ESD) or endoscopic mucosal resection (EMR) between 2013 and 2020 at a single institution.

Results

Ten patients underwent 37 resections by ESD (n=16), EMR (n=15), and snare resection (SR, n=6). For patients with available follow-up endoscopies, the overall recurrence rate was 9/13 (69.2%) for ESD procedures, 14/14 (100%) for EMR procedures, and 2/2 (100%) for SR procedures, with an average follow-up time of 9.4 months. All cases excised by ESD without recurrence had negative deep and lateral margins. The most common adverse event associated with ESD and EMR is bleeding. Histological examination reveals classic features of gastric hyperplasia, including superficial ulceration, granulation tissue with reactive basal atypia, gastropathy and gastritis-like features. There was one case with intestinal metaplasia, yet none revealed dysplasia or neoplasia and all the studied samples were negative for *Helicobacter pylori* organisms by immunostaining.

Conclusions

Large GHPs are clinically challenging and difficult to eradicate with a high rate of recurrence when excised by EMR or SR. ESD appears to be more effective than EMR and SR for complete resection. As the deep margins are almost always negative for ESD specimens, it appears that GHP has a field effect with local recurrence at the lateral margins. Pinning and inking the lateral margins of friable specimens intraoperatively may be helpful for pathologic examination.

## Introduction

Gastric polyps are detected in 0.36% to 6% of patients undergoing upper gastrointestinal endoscopy [1-3]. Gastric hyperplastic polyp (GHP) is the second most common type of gastric polyp and is characterized by hyperplastic, elongated, and dilated foveolae with a variable degree of inflamed stroma. These are detected most often in the sixth or seventh decade of life and are often associated with *Helicobacter pylori* gastritis, autoimmune gastritis, and chemical or reactive gastropathy, and post-surgery [4,5]. The majority of patients are asymptomatic; however, larger GHPs can lead to low-grade bleeding, iron-deficiency anemia, or even gastric outlet obstruction [6].

Dysplasia in GHP is uncommon and the reported incidence varies significantly [5,7,8]. The risk for dysplasia is reported to occur with larger polyp size and older patient age [5,8]. Some guidelines recommend resection of all GHPs >10 mm or >5 mm, but it is not universal [9-11]. Strategies for endoscopic removal of GHP are evolving and R0 resection (complete histologic resection) is preferred to piecemeal polypectomy technique. For large GHPs, endoscopic mucosal resection (EMR) and endoscopic submucosal dissection (ESD) have been used. The purpose of this study is to review our experience with EMR and ESD for the resection of large GHPs and examine the histological findings, adverse events, proportion of recurrence on follow-up endoscopy, and risk factors for recurrence.

## Materials and methods

Study design

**Figure 1:**
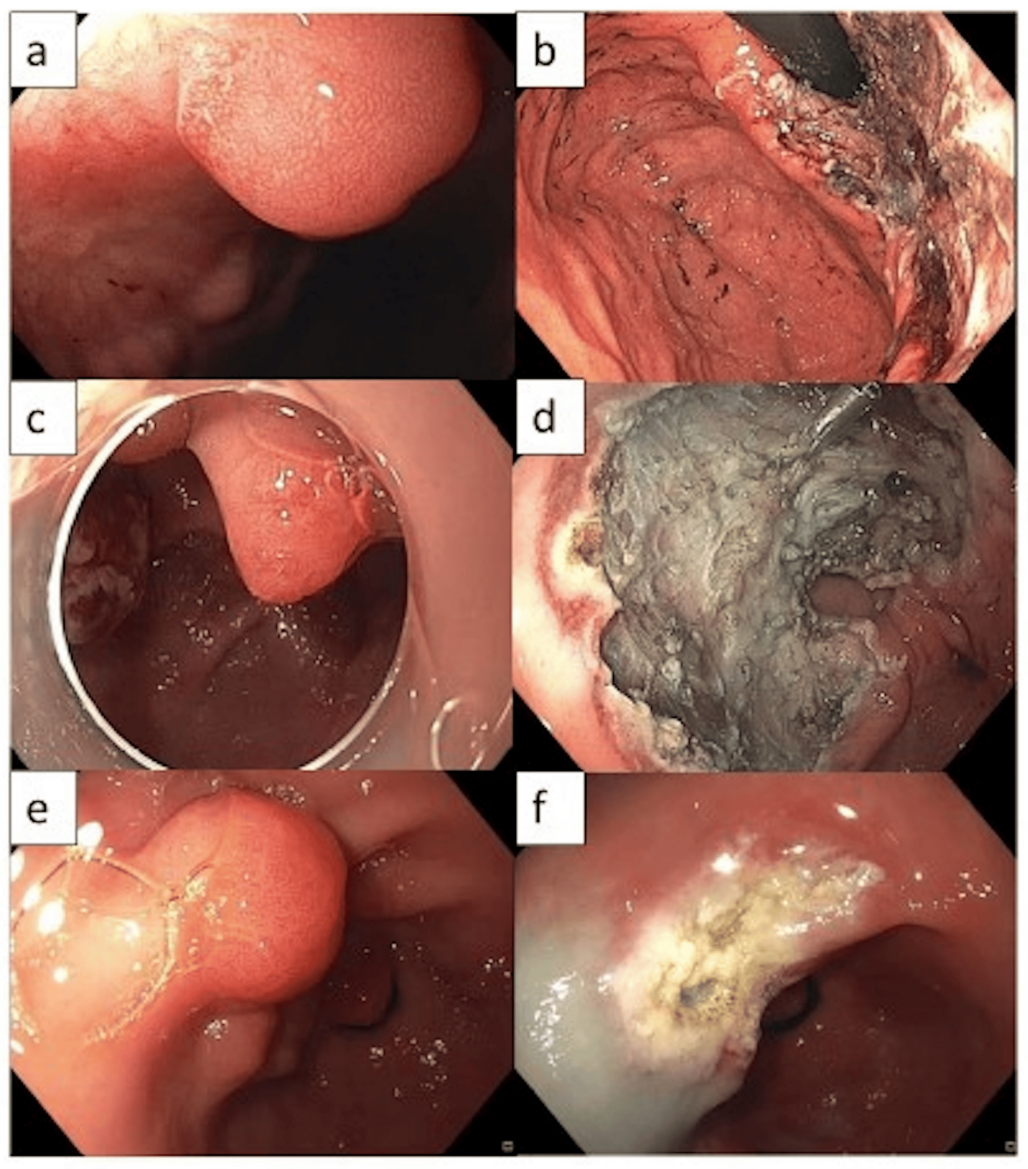
Large GHP of the gastric body pre (a) and post (b) ESD. A recurrent GHP of the antrum pre (c) and post (d) ESD. A large GHP of the antrum pre (e) and post (f) EMR. GHP: Gastric hyperplastic polyp; ESD: endoscopic submucosal dissection; EMR: endoscopic mucosal resection.

This study is a single-center retrospective study. All patients who underwent ESD for the treatment of GHP at Penn State Health Milton S. Hershey Medical Center between 2013 and 2020 were reviewed. Only patients with large GHP (size ≥ 20 mm) were included (Figure 1).

Once identified, the electronic medical record was reviewed for all subsequent endoscopic treatments (ESD, EMR, or SR) to recurrent lesions at the same location. Recurrence was defined as endoscopic appearance with histologic confirmation. The patients’ clinical course, including adverse events or hospitalization within 30 days of the procedure, recurrence and subsequent treatment, was reviewed. The patients’ comorbidity and histology slides, including special stains and immunohistochemical stains, were also reviewed. Patients who received EMR and/or SR treatment only and patients who are <18 years old were excluded from the study.

Objectives

The primary endpoint was the proportion of recurrence on follow-up endoscopy (typically at six months). Secondary endpoints were: (1) proportion of R0 resections; (2) risk factors for recurrence; (3) adverse events or hospitalization within 30 days of the procedure; and (4) clinicopathological and follow-up findings.

Statistics

Descriptive statistics were used to quantitatively summarize the patients’ demographics, clinical course, and pathologic findings related to GHPs. The analysis of variance (ANOVA) test was used for quantitative variables (polyp size, procedure time, and recurrence rate). Statistical analysis was done using MedCalc statistical software (MedCalc Software Ltd., Ostend, Belgium). The level of significance was set at p≤0.05.

Ethics

This study was conducted according to the Declaration of Helsinki and received approval from the Pennsylvania State University Institutional Review Board (study ID 00026677).

## Results

Patients

Between 2013 and 2020, a total of 188 patients underwent ESD for GHP. Among them, 10 patients underwent at least one ESD procedure for the treatment of large GHP. In total, these patients underwent 37 procedures (16 ESD, 15 EMR, and six SR). Seven patients were women and three were men. The average age was 64.8 years (range 42-83 years) and all were Caucasian. The patients’ comorbidities included chronic gastroesophageal reflux disease (GERD) (nine patients), obesity (eight patients), hypertension (seven patients), diabetes mellitus (four patients), hyperlipidemia (four patients), obstructive sleep apnea (three patients), Barrett’s esophagus (three patients), and cirrhosis (two patients). Additionally, eight patients were prescribed daily proton pump inhibitor (PPI), and three patients were on therapeutic anticoagulation. There were no active smokers, but four patients reported previous tobacco use. Eight patients had their initial endoscopy due to dyspepsia or recurrent emesis, and the remaining two patients due to anemia (Table 1).

**Table 1:** Summary of the patients' demographics, comorbidities, smoking status, medications, and the indication for initial endoscopy. GERD: gastroesophageal reflux disease; PPI: proton pump inhibitor.

Patients, n	10
Mean age (in years)	64.8 (range 42-83)
Gender:	
Female	7 (70%)
Male	3 (30%)
Comorbidity and smoking status:	
GERD	9 (90%)
Obesity	8 (80%)
Hypertension	7 (70%)
Diabetes mellitus	4 (40%)
Hyperlipidemia	4 (40%)
Obstructive sleep apnea	3 (30%)
Barrett’s esophagus	3 (30%)
Cirrhosis	2 (20%)
History of smoking	4 (40%)
Medications:	
Daily PPI	8 (80%)
Therapeutic anticoagulation	3 (30%)
Indication for initial endoscopy:	
Dyspepsia or recurrent emesis	8 (80%)
Anemia	2 (20%)

Procedure

On endoscopy, the polyps were soft and tan-pink to hemorrhagic, friable, irregular, either polypoid, sessile, or fungating. The average size was 26 mm (SD 15 mm), with the largest over 80 mm. The average GHP size for ESD, EMR, and SR was 36.8 mm (SD 16 mm), 24.9 mm (SD 11.6 mm), and 14.0 mm (SD 9 mm), respectively (p≤0.05). The majority of the GHPs (80%) were located in the antrum, while the remaining were located in the body and cardia. R0 resection was achieved in 13 of 16 (81.3%) ESD cases and six of 15 (40%) EMR cases, respectively (p>0.05). The average procedure times for ESD, EMR, and SR were 122 min (SD 48 min), 33 min (SD 12 min), and 24 min (SD 6 min), respectively. The procedure time for ESD was significantly longer than EMR and SR (p<0.01).

Adverse events

Within 30 days, no adverse events were recorded for patients treated with SR procedure. In patients treated with EMR, two (13.3%) had associated hospitalizations (both for four days) due to post-procedure bleeding requiring transfusion. Both patients were on warfarin, which was stopped perioperatively. For ESD, in five of the 16 (31.3%) procedures, the patients were hospitalized in <24 hours for observation. One patient was hospitalized for three days for pain; and one patient was hospitalized for seven days due to melena, but no transfusion was required. No patient experienced perforations or required emergency surgery.

Histopathology

**Figure 2:**
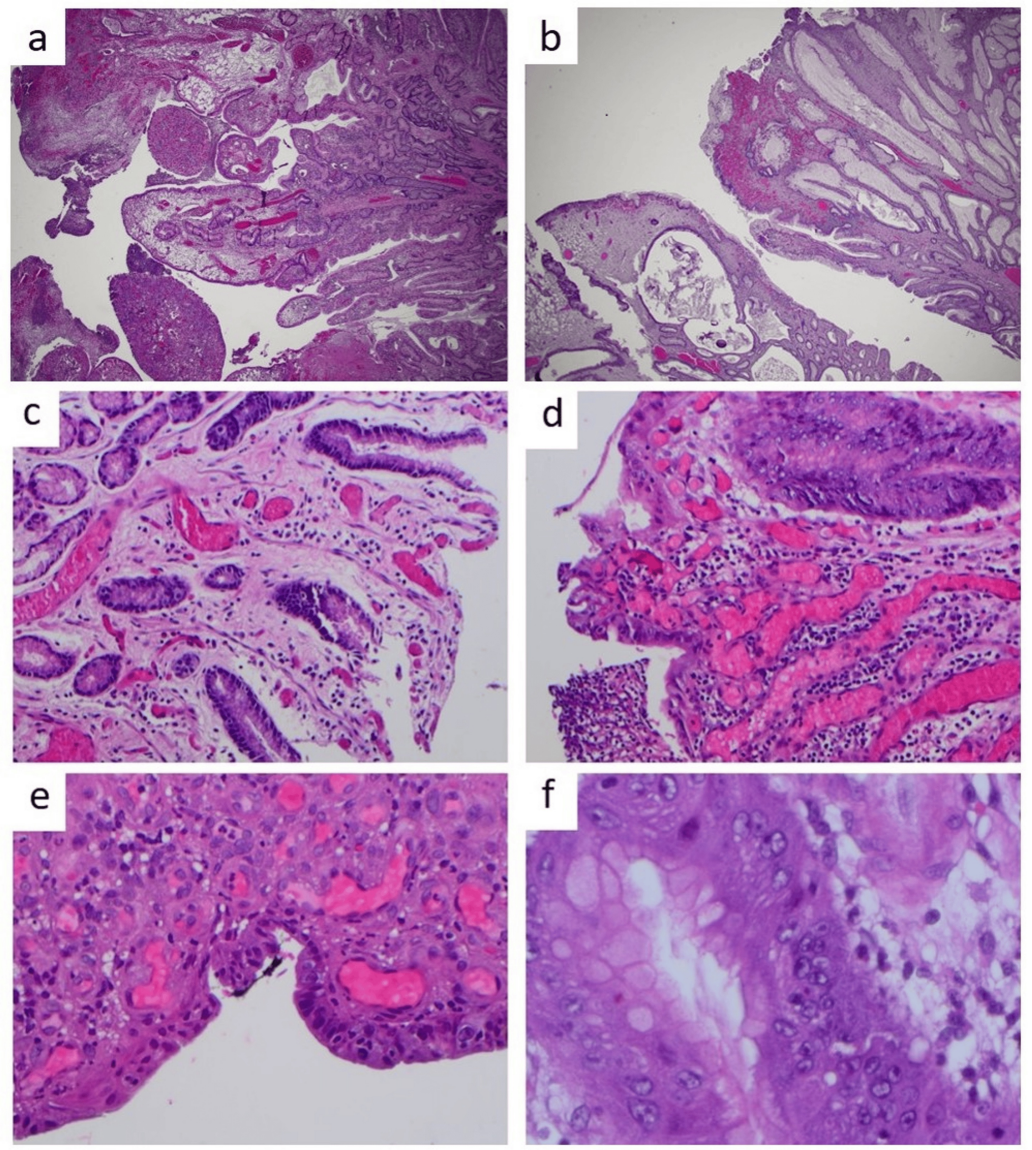
Hematoxylin and eosin (H&E) microphotographs depict a friable, fragmented GHP with ulceration (a, b), reactive changes (c), granulation tissue and reactive nuclear atypia (d, e, f). GHP: Gastric hyperplastic polyp.

Histological examination revealed hyperplastic polyps with superficial ulceration, vascular congestion, and engorgement of superficial granulation tissue vessels, hypertrophic foveolar epithelium, irregular bulbous gastric pits, cystic dilatation of foveolar glands, and stromal mixed inflammation. Typical findings of reactive gastropathy and chronic gastritis (increased lamina propria lymphoplasmacytic inflammation) were observed. There was a histiocytic foreign body granuloma-like aggregate around a gland in one case. Intestinal metaplasia was focally present in one case. Other common histopathological findings included fibrin deposition, thick-walled vessels, and reactive granulation tissue or basilar nuclear atypia (Figure 2).

No dysplasia or malignancy was identified in this case series. Deep resection margins were negative in all cases. Evaluation of lateral resection margins was challenging due to frequent fragmentation of the friable specimens. All cases were negative for *H. pylori*. One case had focal associated neuroendocrine cell hyperplasia without the presence of a neuroendocrine tumor.

Follow-up

Follow-up endoscopy was available for 13 of 16 ESD procedures, 14 of 15 EMR procedures, and two of six SR procedures. The deep margin for the ESD cases was all negative. The overall recurrence rate for ESD procedures was 9/13 procedures (69.2%). Of those 13 ESD procedures, seven were R0 resections and had a recurrence rate of 3/7 (42.9%). The remaining six ESD procedures had piecemeal resection and inadequate lateral margin assessment, and all these patients (100%) had recurrence adjacent to the previous excision site (field effect area). For EMR and SR procedures, the recurrence rate was 14/14 (100%), and 2/2 (100%), respectively (Table 2).

**Table 2:** ESD procedures with R0 resection had the lowest recurrence rate (42.9%). ESD without R0 resection, EMR, and SR procedures all had 100% recurrence. ESD: Endoscopic submucosal dissection; EMR: endoscopic mucosal resection; SR: snare resection.

Procedure	ESD	EMR	SR
Recurrence rate	R0 resections: 3/7 (42.9%)	14/14 (100%)	2/2 (100%)
	Piecemeal resection: 6/6 (100%)		

There is no statistical significance among the groups (p>0.05). The average time to follow-up was 9.4 months (SD 6.2 months).

## Discussion

GHPs are common and most patients are asymptomatic, but it also depends on polyp size and location. When the polyp becomes large, erosion or ulceration of the surface may cause bleeding and anemia. GHPs are clinically challenging to manage and most guidelines recommend removal when they are large. Our results show that ESD with R0 resection has a relatively lower recurrence rate compared to EMR and SR. Tissue friability and fragmentation often makes pathologic evaluation of the lateral margins challenging. All of the deep margins were determined to be negative with the ESD procedure in our series. The recurrence is likely related to positive lateral margins and they are often unable to be thoroughly evaluated given the possible field effect of multifocal GHP. Intraoperative pinning and inking of deep and especially lateral margins of friable ESD specimen may further improve assessment of the lateral edges. Our results support the findings of Forté et al., in that large GHPs recur in over half of the patients initially treated with EMR and ESD [12]. It also reveals that despite repeated, aggressive endoscopic treatments, these lesions tend to persist beyond the initial recurrence.

We analyzed our data regarding conditions that may predict development and recurrence of GHP. Two of our patients carried the diagnosis of cirrhosis. This is consistent with the literature and is thought to be secondary to portal hypertension and resultant disruption of the gastric mucosa [12-14]. Further review of these cirrhotic patients in our study revealed that one patient did have noted gastric portal hypertension and esophageal varices, which have been reported in patients with associated gastric polyps [14]. It is also interesting to note that 80% of our patients were on daily PPI and the rate is comparable to a reported European cohort [12]. Histologically, GHP is characterized by hyperplastic, elongated, and dilated foveolae with inflamed stroma. Superficial irritation, ulceration, and granulation tissue are commonly observed. Reparative/reactive nuclear atypia is often present and should not be misinterpreted as dysplasia. Dysplasia or carcinoma arising from GHP is only rarely reported and it is important not to overcall the reactive granulation tissue or basal atypia that are often observed.

In this study, the size of the GHPs removed by ESD is significantly larger than those removed by EMR and SR, which may contribute to the longer procedure time for ESD. In general, all three procedures appear to be relatively safe. The major adverse event associated with ESD and EMR is bleeding. There were no perforations or need for emergent surgery in our series. This is comparable to previous reports [9,12].

Our study also has limitations. Firstly, we had a limited number of cases in this series. ESD procedures appear to have lower recurrence rate; however, we were not able to reach a statistical significance. Secondly, the endoscopy follow-up of our patients varies. Further studies with larger case number and long-term follow-up will be helpful in this regard.

## Conclusions

In conclusion, large GHPs are mostly benign, yet clinically often recur and are difficult to eradicate, especially with friable lateral margins and clinical field effect. ESD appears to be superior to EMR and SR for the complete excision of large GHP. Pathologic interpretation of margins could be improved by intraoperative pinning/inking, especially of the lateral margins, as the deep margins can be inked and are almost always negative. This clinicopathologic team approach to effective ESD excision would help to improve the pathologic examination and the patients’ outcome.

## References

[1] Carmack SW, Genta RM, Schuler CM, Saboorian MH (2009). The current spectrum of gastric polyps: a 1-year national study of over 120,000 patients. Am J Gastroenterol.

[2] Morais DJ, Yamanaka A, Zeitune JM, Andreollo NA (2007). Gastric polyps: a retrospective analysis of 26,000 digestive endoscopies. Arq Gastroenterol.

[3] Yacoub H, Bibani N, Sabbah M, Bellil N, Ouakaa A, Trad D, Gargouri D (2022). Gastric polyps: a 10-year analysis of 18,496 upper endoscopies. BMC Gastroenterol.

[4] Abraham SC, Singh VK, Yardley JH, Wu TT (2001). Hyperplastic polyps of the stomach: associations with histologic patterns of gastritis and gastric atrophy. Am J Surg Pathol.

[5] Odze Odze, RD RD, Goldblum JR (2022). Surgical Pathology of the GI Tract, Liver, Biliary Tract and Pancreas, 4th ed. Surgical Pathology of the GI Tract, Liver, Biliary Tract and Pancreas, 4th ed..

[6] Jain A, Chaudhary D, Goyal S, Agarwal AK, Sakhuja P (2022). Giant hyperplastic gastric polyp: a diagnostic dilemma!!. Indian J Pathol Microbiol.

[7] João M, Areia M, Alves S (2021). Gastric hyperplastic polyps: a benign entity? Analysis of recurrence and neoplastic transformation in a cohort study. GE Port J Gastroenterol.

[8] Imura J, Hayashi S, Ichikawa K (2014). Malignant transformation of hyperplastic gastric polyps: an immunohistochemical and pathological study of the changes of neoplastic phenotype. Oncol Lett.

[9] Morgan DR, Corral JE, Li D (2025). ACG clinical guideline: diagnosis and management of gastric premalignant conditions. Am J Gastroenterol.

[10] Banks M, Graham D, Jansen M (2019). British Society of Gastroenterology guidelines on the diagnosis and management of patients at risk of gastric adenocarcinoma. Gut.

[11] Evans JA, Chandrasekhara V, Chathadi KV (2015). The role of endoscopy in the management of premalignant and malignant conditions of the stomach. Gastrointest Endosc.

[12] Forté E, Petit B, Walter T (2020). Risk of neoplastic change in large gastric hyperplastic polyps and recurrence after endoscopic resection. Endoscopy.

[13] Martín Domínguez V, Díaz Méndez A, Santander C, García-Buey L (2016). Portal hypertensive polyps, a new entity?. Rev Esp Enferm Dig (Madrid).

[14] Kara D, Hüsing-Kabar A, Schmidt H, Grünewald I, Chandhok G, Maschmeier M, Kabar I (2018). Portal hypertensive polyposis in advanced liver cirrhosis: the unknown entity?. Can J Gastroenterol Hepatol.

